# Characteristics of glucolipid metabolism and complications in novel cluster-based diabetes subgroups: a retrospective study

**DOI:** 10.1186/s12944-023-01953-6

**Published:** 2023-11-21

**Authors:** Xinrong Li, Hui Chen

**Affiliations:** Department of Endocrinology and Metabolism, Lanzhou University Second Hospital, Lanzhou, 730000 Gansu Province China

**Keywords:** Cluster analysis, Complication, Glucolipid metabolism, Insulin function

## Abstract

**Background:**

Glucolipid metabolism plays an important role in the occurrence and development of diabetes mellitus. However, there is limited research on the characteristics of glucolipid metabolism and complications in different subgroups of newly diagnosed diabetes. This study aimed to investigate the characteristics of glucolipid metabolism and complications in novel cluster-based diabetes subgroups and explore the contributions of different glucolipid metabolism indicators to the occurrence of complications and pancreatic function.

**Methods:**

This retrospective study included 547 newly diagnosed type 2 diabetes patients. Age, body mass index (BMI), glycated hemoglobin (HbA_1_C), homeostasis model assessment-2 beta-cell function (HOMA2-β), and homeostasis model assessment-2 insulin resistance (HOMA2-IR) were used as clustering variables. The participants were divided into 4 groups by k-means cluster analysis. The characteristics of glucolipid indicators and complications in each subgroup were analyzed. Regression analyses were used to evaluate the impact of glucolipid metabolism indicators on complications and pancreatic function.

**Results:**

Total cholesterol (TC), triglycerides (TG), triglyceride glucose index (TyG), HbA_1_C, fasting plasma glucose (FPG), and 2-h postprandial plasma glucose (2hPG) were higher in the severe insulin-resistant diabetes (SIRD) and severe insulin-deficient diabetes (SIDD) groups. Fasting insulin (FINS), fasting C-peptide (FCP), 2-h postprandial insulin (2hINS), 2-h postprandial C-peptide (2hCP), and the monocyte-to-high-density lipoprotein cholesterol ratio (MHR) were higher in mild obesity-related diabetes (MOD) and SIRD. 2hCP, FCP, and FINS were positively correlated with HOMA2-β, while FPG, TyG, HbA_1_C, and TG were negatively correlated with HOMA2-β. FINS, FPG, FCP, and HbA_1_C were positively correlated with HOMA2-IR, while high-density lipoprotein (HDL) was negatively correlated with HOMA2-IR. FINS (odds ratio (OR),1.043;95% confidence interval (CI) 1.006 ~ 1.081), FCP (OR,2.881;95%CI 2.041 ~ 4.066), and TyG (OR,1.649;95%CI 1.292 ~ 2.104) contributed to increase the risk of nonalcoholic fatty liver disease (NAFLD); 2hINS (OR,1.015;95%CI 1.008 ~ 1.022) contributed to increase the risk of atherosclerotic cardiovascular disease (ASCVD); FCP (OR,1.297;95%CI 1.027 ~ 1.637) significantly increased the risk of chronic kidney disease (CKD).

**Conclusions:**

There were differences in the characteristics of glucolipid metabolism as well as complications among different subgroups of newly diagnosed type 2 diabetes. 2hCP, FCP, FINS, FPG, TyG, HbA_1_C, HDL and TG influenced the function of insulin. FINS, TyG, 2hINS, and FCP were associated with ASCVD, NAFLD, and CKD in newly diagnosed T2DM patients.

## Introduction

Diabetes mellitus has become the third largest noncommunicable chronic disease after cardiovascular diseases and cancer, severely impacting people's quality of life. In 2021, approximately 537 million adults worldwide had diabetes, and this number is projected to rise to 643 million by 2030 and 784 million by 2045 [1]. Of note, more than 90% of these patients have type 2 diabetes mellitus (T2DM). T2DM is a chronic progressive disease characterized by elevated blood sugar levels and abnormal lipid metabolism [2]. There are 116 million diabetes patients in China. However, only 45% of patients reach the target level for low-density lipoprotein [3]. The rate of achieving the target level for blood lipids is also low in both the United States and Poland [4, 5]. Poor blood glucose control can lead to elevated levels of triglycerides and low-density lipoprotein, as well as a decrease in high-density lipoprotein in diabetes patients [6]. Therefore, it is crucial to study the characteristics of glucolipid metabolism in newly diagnosed T2DM patients, aiming to control and manage the disease and reduce having the risk of complications.

The Ahlqvist team first applied cluster analysis to newly diagnosed diabetes patients. By using variables such as age at diagnosis, body mass index (BMI), glycated hemoglobin (HbA_1_C), homeostasis model assessment-2 of insulin resistance (HOMA2-IR), homeostasis model assessment-2 of beta-cell function (HOMA2-β), and glutamic acid decarboxylase antibody (GADA), they identified five subgroups: severe autoimmune diabetes (SAID), severe insulin-deficient diabetes (SIDD), severe insulin-resistant diabetes (SIRD), mild obesity-related diabetes (MOD), and mild age-related diabetes (MARD) [7]. The SAID subgroup was characterized by a high prevalence of GADA, with poorer pancreatic function. The SIDD subgroup had the highest risk of retinopathy. The SIRD subgroup had the highest HOMA2-IR, with the highest risk of nonalcoholic fatty liver disease (NAFLD) and chronic kidney disease (CKD). Subsequently, several scholars have validated this conclusion and obtained relatively stable classification results, indicating that this method has a better-guiding value for the treatment and prognosis of the disease [8–15]. However, due to the heterogeneity of races, the characteristics and proportions of subgroups may vary among different study populations [7–10, 16–18].

Trajectory analysis indicates that changes in metabolism and inflammation-related biomarkers begin more than 10 years before the onset of T2DM [19–21]. Notably, dyslipidemia has been improved as an important factor in the occurrence and development of T2DM and its complications [22–25]. Dyslipidemia can affect the function of the pancreas and other organs by influencing insulin secretion and peripheral insulin sensitivity. Hyperglycemia can impact the synthesis and breakdown of body fat, leading to further development of insulin resistance, thus forming a vicious cycle of glucose and lipid metabolism. In patients with T2DM, 75% of deaths are attributed to atherosclerotic cardiovascular disease (ASCVD). Dyslipidemia, which accelerates the progression of atherosclerosis, is a primary risk factor for fatal and non-fatal myocardial infarction [26]. The American Diabetes Association recommends that individuals at high risk for cardiovascular disease should receive statin therapy regardless of their baseline lipid levels [27]. Furthermore, during dyslipidemia, excessive fatty acids can enter multiple metabolic pathways, activating signaling molecules associated with other complications of diabetes. This can accelerate the occurrence and development of complications. Clustering subtypes provide a potential approach for precision medicine in diabetes, making it crucial to study the characteristics of glucose and lipid metabolism as well as complications in each subgroup.

However, there is currently limited research on the characteristics of glucolipid metabolism in each subgroup and their relationship with complications. The contribution of various metabolism indicators to complications and pancreatic function is still unclear. This study explored the differences in glucolipid metabolism indicators among the subgroups and analyzed the occurrence of chronic complications. Additionally, the contributions of various glucolipid metabolism indicators to pancreatic function and complications were also discussed separately.

## Methods

### Participants

Cross-sectional descriptive research was conducted on this issue. According to the diagnostic criteria of the World Health Organization [28], a total of 547 newly diagnosed T2DM patients admitted to the Endocrinology and Metabolism Department of Lanzhou University Second Hospital from May 2019 to August 2022 were selected as the study subjects. The exclusion criteria were as follows: (1) Patients who had received regular antidiabetic treatment or regular lipid-lowering treatment; (2) Patients had a disease duration of more than 1 year; (3) Type 1 diabetes, gestational diabetes, and other special types of diabetes; (4) Acute severe complications of diabetes;(5) Patients with severe infections, severe cardiac, hepatic, or renal dysfunction, or tumors; (6) Pregnant and lactating women. The research was approved by the Lanzhou University Second Hospital Ethics Committee (reference number: 2023A-001). All patients enrolled in this study provided informed consent, and the study was conducted ethically by the World Medical Association Declaration of Helsinki.

### Measurements

The data collection for the present study included demographic characteristics, glycemic and lipid metabolism indicators, and the occurrence of complications. Demographic characteristics included age, gender, height, weight, BMI, systolic blood pressure, diastolic blood pressure, history of hypertension, smoking history, and alcohol consumption history. Glycemic and lipid metabolism indicators were measured by using standard methods upon the patient's admission to the hospital, including fasting plasma glucose (FPG), fasting insulin (FINS), fasting C-peptide (FCP), 2-h postprandial plasma glucose (2hPG), 2-h postprandial insulin (2hINS), 2-h postprandial C-peptide (2hCP), HbA_1_C, total cholesterol (TC), triglycerides (TG), triglyceride glucose index (TyG), high-density lipoprotein (HDL), low-density lipoprotein (LDL) and monocyte/high-density lipoprotein ratio (MHR). The OGTT test (Oral Glucose Tolerance Test), which involved measuring a glucose load of 75 g, was used to measure blood sugar levels and indicators related to pancreatic function, such as FPG (fasting plasma glucose), 2hPG (2-h postprandial glucose), FCP (fasting C-peptide), 2hCP (2-h postprandial C-peptide), FINS (fasting insulin level), and 2hINS (2-h postprandial insulin level). Apart from that, the other indicators were obtained from the venous blood collected in the antecubital fossa after the patient fasted for more than 8 h overnight, and the serum was separated at room temperature. All indicators were measured by a Hitachi 7600-DDP fully automatic biochemical analyzer (LABOSPECT, Japan) in our hospital's laboratory. Insulin determination was performed using electrochemiluminescence immunoassay (SIEMENS, Germany), and HbA_1_C determination was performed using high-performance liquid chromatography (Burler-D10, America). The HOMA-related indices were calculated by the Oxford University HOMA2 calculator [29]. The triglyceride glucose (TyG) index was defined as ln (fasting TG (mg/dL) × FPG (mg/dL)/2) [30]. The data collection process utilized standardized equipment and procedures. The measurements of the same indicators were carried out using the same model of equipment, ensuring the use of consistent measurement procedures and steps in each instance. Operators had received training to ensure the correct execution of the measurement procedures.

Complications were defined based on the ICD-10 codes (diabetic peripheral neuropathy (DPN) E11.4; CKD E11.2; diabetic retinopathy (DR) E11.5; peripheral vascular disease (PVD) I73; ASCVD (coronary artery disease, stroke, and/or peripheral artery disease, ascertained by self-report or any of the following ICD-10 codes: I20-I25, I63, I64, G45, E11.5, and I73.9 respectively); NAFLD K76.0 or on the original diagnosis text of the physicians.

### Cluster analysis

Based on the study of Ahlqvist et al. [7], age, HbA_1_C, BMI, HOMA2-β, and HOMA2-IR were used as cluster variables for cluster analysis. The variables were standardized before clustering. Patients with extreme outliers (mean > 5 standard deviations) were excluded. K-means clustering was performed with a k value of 4 in SPSS 25.0.

### Statistical analysis

Analyses were conducted by SPSS 25.0 software (IBM Corp., Armonk, NY, United States). Non-normally distributed data were transformed using logarithmic conversion before clustering. Missing values were less than 5% and were imputed using the median or mode. Continuous variables that followed a normal distribution were presented as mean ± standard deviation, while non-normally distributed continuous variables were presented as median and interquartile range. All categorical variables were represented as numbers (proportions). Group comparisons of continuous variables were performed using one-way ANOVA or the Mann–Whitney U test. When comparing pairwise between groups, the least significant difference method was utilized. Group comparisons of categorical variables were conducted using the chi-square test. For conducting multiple comparisons of proportions among multiple groups, the Bonferroni correction was applied to adjust the significance level. Spearman correlation analysis was used to investigate the correlation between pancreatic function and glycemic and lipid metabolism indicators. Binary stepwise logistic regression analysis and multiple stepwise linear regression analysis were utilized to analyze the factors that influence the independent variables. The forward selection method was employed for regression analysis, with the first group being used as the reference category.

## Results

### Cluster groups and demographic characteristics

**Fig. 1:**
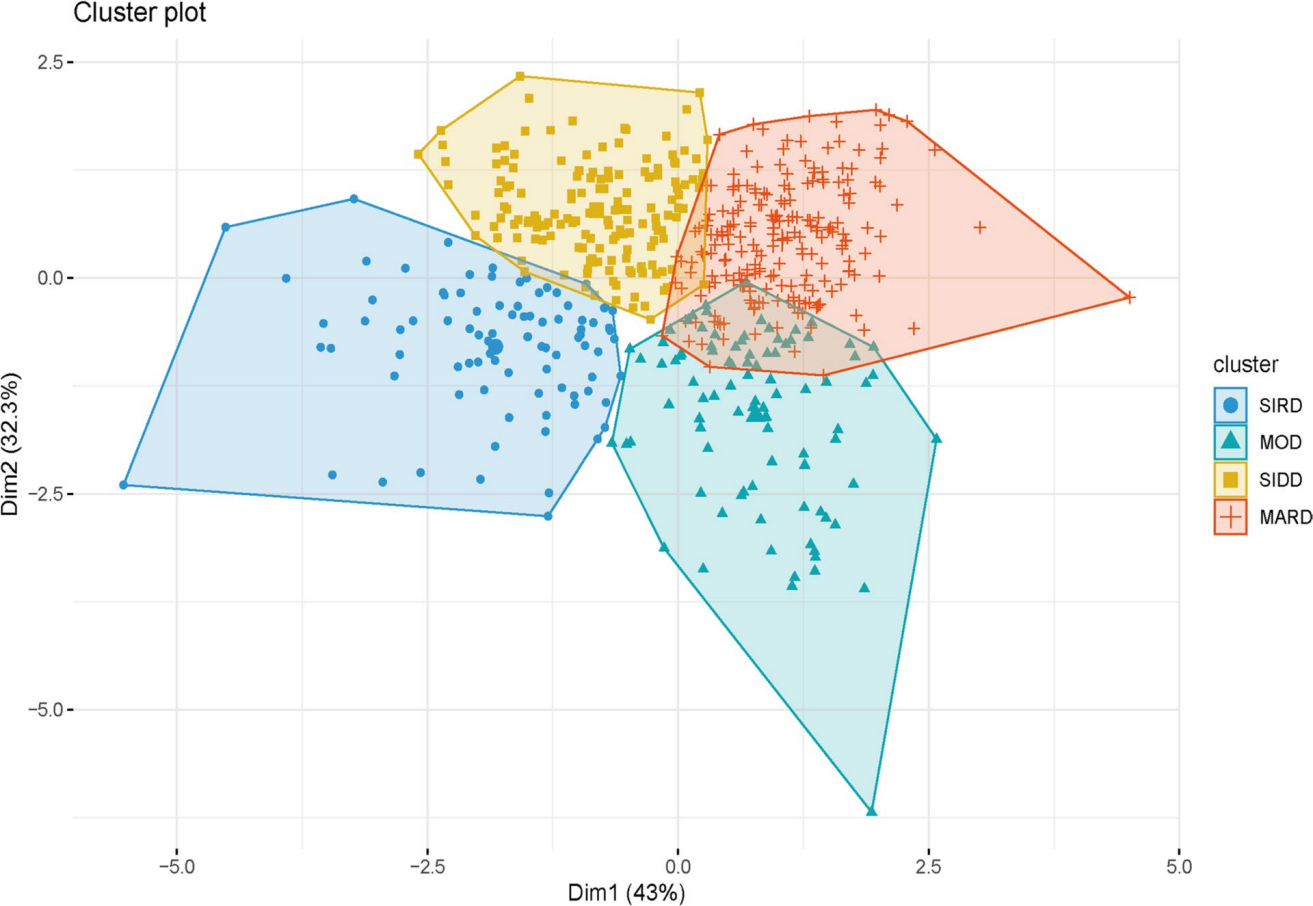
Cluster analysis results for newly diagnosed type 2 diabetes patients. The k-means clustering method was used and k = 4. *Abbreviations*: SIDD, severe insulin-deficient diabetes; SIRD, severe insulin-resistant diabetes; MOD, mild obesity-related diabetes; MARD, mild age-related diabetes

A total of 547 patients with newly diagnosed T2DM were ultimately included in this study. As shown in Fig. 1, the research subjects were clustered into four subgroups based on age, BMI, HbA_1_C, HOMA2-β, and HOMA2-IR, which were used as clustering variables. The four groups were SIDD, SIRD, MOD, and MARD. In the clustering analysis, the MARD group had the highest proportion of individuals, with 168 people (30.71%), followed by 141 people (25.78%) in the SIDD group and 136 people (24.86%) in the MOD group. The SIRD group had the fewest individuals, with 102 people (18.65%).

Table 1 illustrated the demographic characteristics of the cluster groups. There was statistical significance in terms of the history of hypertension, diastolic blood pressure, BMI, and weight differences when comparing the overall population (*P* < 0.05). When conducting pairwise comparisons between groups, the MOD group had the highest number of people with a history of hypertension, while the SIRD group had the lowest number of people with a history of hypertension. The MOD group had the highest diastolic blood pressure, while the SIDD group had the lowest diastolic blood pressure. The MOD group had the highest weight and BMI, while the SIDD group had the lowest weight and BMI (*P* < 0.05).

**Table 1:** The demographic characteristics of subgroups

Variables	MARD(*n* = 168)	MOD(*n* = 136)	SIRD(*n* = 102)	SIDD(*n* = 141)	*P*
Male n(%)	119(70.83%)	85(62.50%)	32(31.37%)	99(70.21%)	0.748
Smoking n(%)	41(24.40%)	36(26.47%)	23(22.55%)	42(29.79%)	0.624
Alcohol n(%)	16(9.52%)	14(10.29%)	5(4.90%)	15(10.64%)	0.401
Hypertension n(%)	47(27.98%)	50(36.76%)^a^	24(23.53%)^b^	27(19.15%)^abc^	**0.003**
Age (years)	51.10 ± 0.96	53.71 ± 0.89	53.16 ± 1.37	52.94 ± 1.10	0.301
SBP (mmHg)	129(116,145)	130(119.143)	126(113,141)	126(112,139)	0.138
DBP (mmHg)	80(73,88)	85(78,93)^a^	84(75,92)	79(74,87)^b^	**0.004**
Height (cm)	169(162,174)	170(160,175)	170(164,175)	170(163,175)	0.549
Weight (kg)	68.0(60.1,75.0)	75.0(66.0,85.0)^a^	72.5(65.0,82.0)^a^	68.5(60.0,75.0)^bc^	** < 0.001**
BMI	24.0(21.9,25.7)	26.8(24.1,29.3)^a^	25.4(23.8,27.8)^a^	23.8(21.5,26.1)^bc^	** < 0.001**

Continuous variables that follow a Gaussian distribution are presented as the mean (standard deviation), while skewed distributed continuous variables are presented as the median (interquartile range). All categorical variables were represented by numbers or proportions. Group comparisons of continuous variables were performed using ANOVA or the Mann–Whitney U test. When conducting pairwise comparisons between groups, the least significant difference method is used. Group comparisons of categorical variables were conducted using the chi-square test. For multiple comparisons of proportions among multiple groups, Bonferroni correction is used to adjust the significance level. The difference is considered statistically significant when *P* < 0.05

*Abbreviations*: *SIDD* severe insulin-deficient diabetes, *SIRD* severe insulin-resistant diabetes, *MOD* mild obesity-related diabetes, *MARD* mild age-related diabetes, *BMI* body mass index, *SBP* systolic blood pressure, *DBP* diastolic blood pressure

^a^*p* < 0.05 represents a comparison of MOD, SIRD, and SIDD groups with the MARD group

^b^*p* < 0.05 represents a comparison of SIRD and SIDD groups with the MOD group

^c^*p* < 0.05 represents a comparison of the SIDD group with the SIRD group

### Characteristics of glucolipid metabolism and complications in each subgroup

**Fig. 2:**
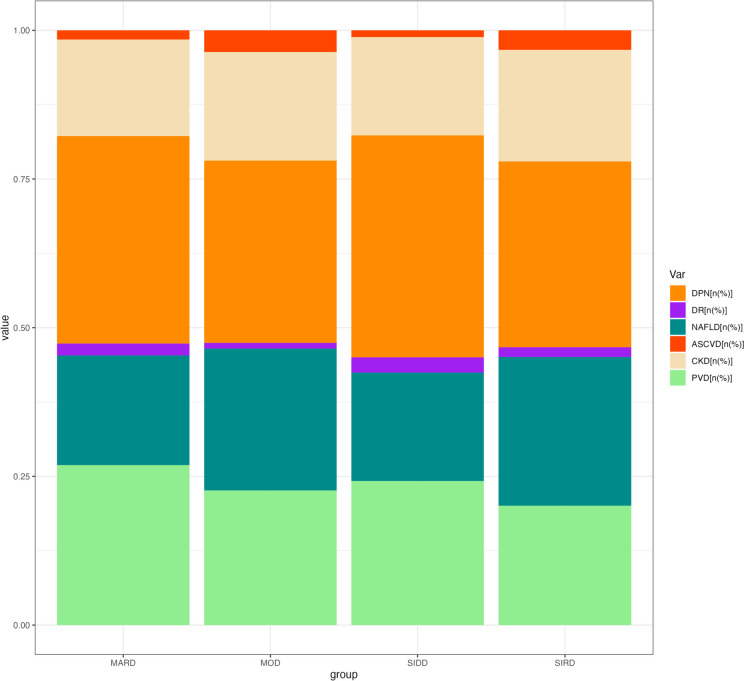
Complications of each subgroup. The stacked bar chart was conducted based on percentages. *Abbreviations*: DPN, diabetic peripheral neuropathy; DR, diabetic retinopathy; NAFLD, non-alcohol Fatty Liver Disease; ASCVD, arteriosclerotic cardiovascular disease; CKD, chronic kidney disease; PVD, peripheral vascular disease

Table 2 (at the end of the manuscript) showed the difference in glucolipid metabolism and the incidence rates of complications among the subgroups. TC, TG, TyG, HbA_1_C, FPG and 2hPG in SIRD and SIDD groups were significantly higher than those in MOD and MARD groups. In addition, FINS, FCP, 2hINS, 2hCP, and MHR were higher in MOD and SIRD, especially in the MOD group. In the SIDD group, FINS, FCP, 2hINS, 2hCP, and MHR were the lowest, while LDL was relatively higher. In the SIRD group, HDL was the lowest while TG/HDL was the highest. As shown in Fig. 2, the highest rates of NAFLD and CKD were observed in the SIRD group, while ASCVD had the highest incidence rate in the MOD group (*P* < 0.05). There was no statistically significant difference in the incidence rates of DPN, DR, and PVD among the groups (*P* > 0.05).

**Table 2:** Basic characteristics in subgroups of newly diagnosed T2DM

Variables	MARD	MOD	SIRD	SIDD	*P*
TC (mmol/L)	4.42(3.73,4.97)	4.31(3.73,4.99)	4.57(3.92,5.52)	4.87(4.14,5.53)^ab^	** < 0.001**
TG (mmol/L)	1.59(1.12,2.38)	1.69(1.33,2.39)	2.63(1.53,4.04)^ab^	1.85(1.33,2.95)^ac^	** < 0.001**
LDL (mmol/L)	2.94(2.41,3.51)	3.01(2.45,3.52)	2.99(2.54,3.67)	3.23(2.72,3.84)^ab^	0.009
HDL (mmol/L)	1.09(0.96,1.30)	1.08(0.92,1.28)	0.99(0.81,1.20)^a^	1.14(0.95,1.43)^c^	** < 0.001**
TyG	7.65(7.24,8.15)	7.56(7.30,7.90)	8.77(8.14,9.23)^ab^	8.31(7.95,8.78)^ab^	** < 0.001**
TG/HDL	1.30(0.89,2.37)	1.52(1.12,2.31)	2.34(1.45,4.55)^ab^	1.55(1.04,2.69)^c^	** < 0.001**
HbA1C(%)	8.51(7.20,10.32)	7.32(6.51,8.42)^a^	11.42(10.15,12.41)^ab^	11.61(10.62,12.81)^ab^	** < 0.001**
FPG (mmol/L)	8.41(7.19,9.72)	7.04(6.43,8.25)^a^	14.28(11.91,17.18)^ab^	13.81(12.12,16.16)^ab^	** < 0.001**
FINS (mU/L)	6.77(4.49,8.95)	13.17(10.62,16.66)^a^	13.72(10.87,17.19)^a^	5.65(3.89,7.33)^bc^	** < 0.001**
FCP (ng/ml)	1.35(1.04,1.75)	2.26(1.88,2.62)^a^	1.99(1.13,2.45)^ab^	1.16(0.86,1.55)^bc^	** < 0.001**
2hPG (mmol/L)	10.64(8.21,14.35)	10.80(8.27,13.17)	14.11(8.87,18.72)^ab^	12.43(9.32,17.90)^ab^	** < 0.001**
2hINS (mU/L)	28.57(16.87,42.11)	53.25(33.24,84.34)^a^	42.39(26.44,57.89)^ab^	30.85(13.29,45.54)^bc^	** < 0.001**
2hCP (ng/ml)	3.43(2.31,4.94)	6.22(4.49,7.99)^a^	2.82(1.93,3.78)^b^	1.96(1.32,2.65)^abc^	** < 0.001**
MHR	0.33(0.26,0.45)	0.36(0.28,0.46)	0.40(0.28,0.53)^a^	0.31(0.23,0.43)^cb^	** < 0.001**
DPN n(%)	157(93.51%)	126(92.63%)	95(93.14%)	131(92.92%)	0.994
DR n(%)	9(5.41%)	4(2.92%)	5(4.93%)	9(6.42%)	0.605
NAFLD n(%)	83(49.42%)	98(72.14%)^a^	76(74.53%)^a^	64(45.45%)^bc^	** < 0.001**
ASCVD n(%)	7(4.23%)	15(11.04%)	10(9.82%)	4(2.83%)^b^	**0.012**
CKD n(%)	73(43.54%)	75(55.13%)	57(55.92%)	58(41.13%)	**0.025**
PVD n(%)	121(72.00%)	93(68.44%)	61(59.85%)	85(60.34%)	0.077

Continuous variables that follow a Gaussian distribution are presented as the mean (standard deviation), while skewed distributed continuous variables are presented as the median (interquartile range). All categorical variables were represented by numbers or proportions. Group comparisons of continuous variables were performed using ANOVA or the Mann–Whitney U test. When comparing pairwise, the least significant difference method is used. Group comparisons of categorical variables were conducted using the chi-square test. For multiple comparisons of proportions among multiple groups, Bonferroni correction is used to adjust the significance level. When *p* < 0.05, the difference is considered statistically significant

*Abbreviations*: *TC* total cholesterol, *TG* triglycerides, *LDL* low-density lipoprotein, *HDL* high-density lipoprotein, *TyG* triglyceride glucose index, *TG/HDL* triglycerides to high-density lipoprotein ratio, *MHR* monocyte-to-high-density lipoprotein cholesterol ratio, *HbA*_*1*_*C* glycated hemoglobin, *FPG* fasting plasma glucose, *FINS* fasting insulin, *FCP* fasting C-peptide, *2hPG* 2-h postprandial plasma glucose, *2hINS* 2-h postprandial insulin, *2hCP* 2-h postprandial C-peptide, *DPN* diabetic peripheral neuropathy, *DR* diabetic retinopathy, *NAFLD* non-alcohol Fatty Liver Disease, *ASCVD* arteriosclerotic cardiovascular disease, *CKD* chronic kidney disease, *PVD* peripheral vascular disease

^a^*p* < 0.05 represents a comparison of MOD, SIRD, and SIDD groups with the MARD group

^b^*p* < 0.05 represents a comparison of SIRD and SIDD groups with the MOD group

^c^*p* < 0.05 represents a comparison of the SIDD group with the SIRD group

### Association of glucolipid metabolism indicators with pancreatic function

The pancreatic function was assessed using the HOMA index, which included HOMA2-β and HOMA2-IR. Table 3 performed the correlation analysis between pancreatic function indicators and glucolipid metabolism indicators. Multiple linear regression analyses showed that FINS, FCP, and 2hCP were positively correlated with HOMA2-β, while FPG, TyG, HbA_1_C, and TG were negatively correlated with HOMA2-β. FINS, FPG, FCP, and HbA_1_C were positively correlated with HOMA2-IR, while HDL was negatively correlated with HOMA2-IR (Table 4).

**Table 3:** Spearman correlation analysis between glucolipid metabolism and pancreatic islet function

Variables	HOMA2-β		HOMA2-IR	
	***r***_***s***_	***p***	***r***_***s***_	***p***
TC(mmol/L)	-0.191	** < 0.001**	0.046	0.283
TG(mmol/L)	-0.169	** < 0.001**	0.265	** < 0.001**
LDL(mmol/L)	-0.146	**0.001**	0.032	0.453
HDL(mmol/L)	0.021	0.624	-0.193	** < 0.001**
TyG	-0.557	** < 0.001**	0.318	** < 0.001**
TG/HDL	-0.109	** < 0.001**	0.283	** < 0.001**
MHR	0.069	0.107	0.123	**0.004**
HbA_1_C(%)	-0.739	** < 0.001**	0.166	** < 0.001**
FPG(mmol/L)	-0.886	** < 0.001**	0.283	** < 0.001**
FINS(mU/L)	0.348	** < 0.001**	0.796	** < 0.001**
FCP(ng/ml)	0.471	** < 0.001**	0.499	** < 0.001**
2hPG(mmol/L)	-0.254	** < 0.001**	0.133	**0.002**
2hINS(mU/L)	0.283	** < 0.001**	0.334	** < 0.001**
2hCP(ng/ml)	0.695	** < 0.001**	0.129	**0.002**

Spearman correlation analysis was used to study the relationship between glucolipid metabolism indicators and pancreatic function

*Abbreviations: TC* total cholesterol; TG, triglycerides, *LDL* low-density lipoprotein, *HDL* high-density lipoprotein, *TyG* triglyceride glucose index, *TG/HDL* triglycerides to high-density lipoprotein ratio, *MHR* monocyte-to-high-density lipoprotein cholesterol ratio, *HbA*_*1*_*C* glycated hemoglobin, *FPG* fasting plasma glucose, *FINS* fasting insulin, *FCP* fasting C-peptide, *2hPG* 2-h postprandial plasma glucose, *2hINS* 2-h postprandial insulin, *2hCP* 2-h postprandial C-peptide

**Table 4:** Stepwise multiple linear regression analyses of glucolipid metabolism associated with pancreatic islet function

Variables	B	SE	Beta	*P*	95%CI
**HOMA2-β**					
FPG(mmol/L)	-2.665	0.307	-0.373	** < 0.001**	-3.268 ~ -2.061
2hCP(ng/ml)	2.503	0.337	0.238	** < 0.001**	1.842 ~ 3.164
FCP(ng/ml)	8.391	1.208	0.211	** < 0.001**	6.018 ~ 10.765
TyG	-7.794	1.904	-0.224	** < 0.001**	-11.534 ~ -4.055
FINS(mU/L)	0.383	0.108	0.095	** < 0.001**	0.172 ~ 0.595
HbA_1_C(%)	-1.195	0.410	-0.103	**0.004**	-2.001 ~ -0.390
TG(mmol/L)	-1.261	0.480	-0.109	**0.009**	-2.205 ~ -0.318
**HOMA2-IR**					
FINS(mU/L)	0.096	0.006	0.553	** < 0.001**	0.084 ~ 0.108
FPG(mmol/L)	0.151	0.013	0.492	** < 0.001**	0.125 ~ 0.177
FCP(ng/ml)	0.197	0.061	0.116	**0.001**	0.078 ~ 0.316
HbA_1_C(%)	0.057	0.022	0.113	**0.011**	0.013 ~ 0.100
HDL(mmol/L)	-0.180	0.072	-0.074	**0.012**	-0.320 ~ -0.039

*Abbreviations: HOMA2-β* homeostasis model assessment-2 beta-cell function, *FPG* fasting plasma glucose, *2hCP* 2-h postprandial C-peptide, *FCP* fasting C-peptide, *TyG* triglyceride glucose index, *FINS* fasting insulin, *HbA*_*1*_*C* glycated hemoglobin, *TG* triglycerides, *HOMA-2-IR* homeostasis model assessment-2 insulin resistance, *HDL* high-density lipoprotein

### Association of glucolipid metabolism with complications

Both the univariate and multivariate logistic analyses of NAFLD, ASCVD, and CKD were summarized in Table 5 (at the end of the manuscript). After adjusting for other factors, FINS (OR,1.043;95% CI 1.006 ~ 1.081; *p* = 0.021), FCP (OR,2.881;95%CI 2.041 ~ 4.066; *p* < 0.001) and TyG (OR,1.649;95%CI 1.292 ~ 2.104; *p* < 0.001) were associated with increased risk of NAFLD; 2hINS (OR,1.015;95%CI 1.008 ~ 1.022; *p* < 0.001) was associated with increased risk of ASCVD; FCP (OR,1.297;95%CI 1.027 ~ 1.637;* p* = 0.029) was associated with increased risk of CKD. When FINS, FCP, and TyG increased by one unit each, the risk of developing NAFLD increased by 1.043, 2.881, and 1.649 times respectively. When 2hINS increased by one unit, the risk of developing ASCVD increased by 1.015 times. When FCP increased by one unit, the risk of developing CKD increased by 1.297 times.

**Table 5:** Univariate and multiple stepwise linear regression analysis for analyzing glucolipid metabolism associated with complications

Variables	Univariate		Multivariate	
	**OR(95%CI)**	***P***	**OR(95%CI)**	***P***
**NAFLD**				
HDL(mmol/L)	0.751(0.540 ~ 1.046)	0.091		
MHR	2.749(1.012 ~ 7.555)	**0.049**		
TG/HDL	1.239(1.121 ~ 1.371)	**0.000**		
FPG(mmol/L)	1.005(0.963 ~ 1.047)	0.833		
HbA_1_C(%)	1.046(0.977 ~ 1.120)	0.200		
FINS(mU/L)	1.123(1.082 ~ 1.165)	**0.000**	1.043(1.006 ~ 1.081)	**0.021**
FCP(ng/ml)	3.331(2.466 ~ 4.500)	**0.000**	2.881(2.041 ~ 4.066)	** < 0.001**
HOMA2-β	1.004(0.998 ~ 1.010)	0.215		
HOMA2-IR	1.669(1.378 ~ 2.022)	**0.000**		
2hINS(mU/L)	1.007(1.002 ~ 1.013)	**0.012**		
2hCP(ng/ml)	1.109(1.035 ~ 1.189)	**0.003**		
2hPG(mmol/L)	1.013(0.983 ~ 1.044)	0.402		
TC(mmol/L)	1.260(1.079 ~ 1.471)	**0.003**		
TG(mmol/L)	1.288(1.145 ~ 1.449)	**0.000**		
TyG	1.677(1.344 ~ 2.093)	**0.000**	1.649(1.292 ~ 2.104)	** < 0.001**
LDL(mmol/L)	1.290(1.064 ~ 1.564)	**0.010**		
**ASCVD**				
HDL(mmol/L)	0.949(0.480 ~ 1.875)	0.880		
MHR	0.865(0.121 ~ 6.155)	0.884		
TG/HDL	0.872(0.711 ~ 1.068)	0.185		
FPG(mmol/L)	0.911(0.829 ~ 1.002)	0.055		
HbA_1_C(%)	0.849(0.733 ~ 0.984)	**0.030**		
FINS(mU/L)	1.032(0.996 ~ 1.070)	0.080		
FCP(ng/ml)	1.578(1.051 ~ 2.370)	**0.028**		
HOMA2-β	1.014(1.005 ~ 1.023)	**0.003**		
HOMA2-IR	1.164(0.930 ~ 1.456)	0.185		
2hINS(mU/L)	1.015(1.008 ~ 1.022)	**0.000**	1.015(1.008 ~ 1.022)	** < 0.001**
2hCP(ng/ml)	1.218(1.109 ~ 1.338)	**0.000**		
2hPG(mmol/L)	1.009(0.952 ~ 1.069)	0.769		
TC(mmol/L)	0.866(0.637 ~ 1.176)	0.357		
TG(mmol/L)	0.833(0.650 ~ 1.069)	0.769		
TyG	0.700(0.565 ~ 1.080)	0.107		
LDL(mmol/L)	0.942(0.648 ~ 1.370)	0.755		
**CKD**				
HDL(mmol/L)	0.832(0.597 ~ 1.162)	0.281		
MHR	1.044(0.399 ~ 2.732)	0.929		
TG/HDL	1.030(0.980 ~ 1.082)	0.251		
FPG(mmol/L)	1.013(0.972 ~ 1.056)	0.536		
HbA_1_C(%)	1.028(0.962 ~ 1.100)	0.414		
FINS(mU/L)	1.018(0.994 ~ 1.042)	0.153		
FCP(ng/ml)	1.297(1.027 ~ 1.637)	**0.029**	1.297(1.027 ~ 1.637)	**0.029**
HOMA2-β	0.998(0.992 ~ 1.004)	0.492		
HOMA2-IR	1.113(0.971 ~ 1.276)	0.123		
2hINS(mU/L)	1.001(0.996 ~ 1.006)	0.616		
2hCP(ng/ml)	0.982(0.924 ~ 1.044)	0.563		
2hPG(mmol/L)	1.007(0.978 ~ 1.037)	0.651		
TC(mmol/L)	1.001(0.868 ~ 1.155)	0.987		
TG(mmol/L)	1.055(0.981 ~ 1.134)	0.147		
TyG	1.221(0.997 ~ 1.494)	0.054		
LDL(mmol/L)	1.011(0.841 ~ 1.215)	0.906		

*Abbreviations: TC* total cholesterol, *TG* triglycerides, *LDL* low-density lipoprotein, *HDL* high-density lipoprotein, *TyG* triglyceride glucose index, *TG/HDL* triglycerides to high-density lipoprotein ratio, *MHR* monocyte-to-high-density lipoprotein cholesterol ratio, *HbA*_*1*_*C* glycated hemoglobin, *FPG* fasting plasma glucose, *FINS* fasting insulin, *FCP* fasting C-peptide, *2hPG* 2-h postprandial plasma glucose, *2hINS* 2-h postprandial insulin, *2hCP* 2-h postprandial C-peptide, *DPN* diabetic peripheral neuropathy, *DR* diabetic retinopathy, *NAFLD* non-alcohol Fatty Liver Disease, *ASCVD* arteriosclerotic cardiovascular disease, *CKD* chronic kidney disease, *PVD* peripheral vascular disease

## Discussion

This cross-sectional research revealed the different glucolipid metabolism characteristics among subgroups of newly diagnosed T2DM patients. These characteristics were associated with varying incidence rates of complications in different subgroups. Different glucolipid metabolism indicators also contributed differently to pancreatic function and complications.

It has been confirmed that triglycerides, inflammatory processes, and insulin resistance are significantly related in different populations [31, 32]. As seen in our study, the SIRD group of newly diagnosed T2DM was found to be more prone to experiencing dyslipidemia [16, 33, 34]. In addition, it was shown in the German diabetes cohort that both MOD and SIRD groups had the highest levels of inflammatory mediators at baseline and during the 5-year follow-up, which was supported by Dennis et al. [12] and Herder et al. [35], suggesting a close association between inflammation and insulin resistance [9]. The previous research findings were consistent with the conclusions of our study. In this study, the SIRD group and SIDD group had higher levels of HbA_1_C, FPG, and 2hPG compared to the MARD group and the MOD group, indicating poorer glycemic control. The SIDD group had the lowest HOMA2-β index, as well as lower levels of insulin and C-peptide, suggesting impaired pancreatic beta-cell function. On the other hand, the MOD group had the highest HOMA2-β index and higher levels of insulin and C-peptide, indicating better pancreatic beta-cell function. MHR is a novel marker of inflammation that combines both inflammatory and anti-inflammatory factors. It reflects the inflammatory status and severity of the body better than a single inflammatory marker. It was demonstrated to be the highest in the SIRD group in our study. In terms of lipid metabolism, the SIRD group had the lowest HDL levels and the highest levels of TG/HDL, TC, TG, and TyG, indicating a more dyslipidemia profile. Previous studies have shown that TG/HDL can serve as a marker of insulin resistance in prediabetes [36]. It has also been found that TyG is significantly associated with insulin resistance measured by clamp tests and is closely related to T2DM and NAFLD [37, 38].

Due to poor compliance and inadequate control of blood glucose and other metabolic targets, there is a higher risk of complications in individuals with diabetes [39]. It remains a challenge for both patients and healthcare professionals to further reduce the risk of diabetes-related complications. Indeed, the emergence of clustering analysis methods has provided possibilities for early prevention and treatment of complications. Ahlqvist et al. [7] found that the SIRD group had the highest risk of kidney disease [7], which was consistent with the findings of other researchers [12, 16, 33]. Insulin resistance was associated with impaired kidney function [40]. In the general population of India, it has been found that insulin resistance combined with the absence of the obesity group and the insulin resistance combined with the obesity group have a higher risk of kidney disease [34]. The high risk of kidney disease in the SIRD group suggests that insulin resistance plays a more significant role in the development and early progression of diabetic nephropathy. The risk of cardiovascular diseases in patients with diabetes is 2 ~ 4 times higher than in non-diabetic individuals [41]. Ahlqvist et al. [7, 16] found no difference in the risk of coronary artery disease and stroke among the groups. However, Saatmann et al. [42] proposed that within one year after the diagnosis of diabetes, new subgroups of diabetes had shown significant differences in cardiovascular risk: the SAID group had a lower risk of cardiovascular disease, while the SIRD subgroup had a higher risk, consistent with the findings of Fedotkina et al. [14] and Tanabe et al. [12]. Kahkoska et al. [43] found that the SIDD group had the highest risk of cardiovascular and all-cause mortality, while the MOD group had the highest risk of heart failure. On the other hand, Safai et al. [8] had a different viewpoint and suggested that the presence of cardiovascular disease was not the sole characteristic of the subgroups but rather determined by factors such as age and duration of diabetes. It was reported in our study that the MOD group had the highest prevalence of ASCVD. The possible causes of these differences could be attributed to the heterogeneity of the research population in terms of size and racial diversity. In a 5-year follow-up study conducted in Germany, it was found that the SIRD group had the highest liver cell lipid content at baseline, and liver fibrosis was more common after 5 years [9]. Xing et al. [33], Xiong et al. [16], and Anjana et al. [34] suggested that the SIRD group had poor lipid profiles, which made them more prone to developing fatty liver, which was confirmed by our study. The release of inflammatory processes and inflammatory biomarkers into the bloodstream has been acknowledged as a significant mechanism that can trigger abnormal hepatic glucose metabolism and T2DM [44].

There is a positive correlation between serum inflammatory biomarkers, lipid metabolism, and the occurrence and progression of complications in diabetic patients [45–47]. When there is a disruption in lipid metabolism, adipocytes primarily compensate through hyperplasia and hypertrophy, leading to abnormal adipocyte metabolism and activation of immune cells. This imbalance of adipokines increases the release of inflammatory factors and fatty acids [48]. Fatty acids serve as the foundation for the occurrence and development of DN, DR, and DPN. Excessive fatty acids can enter multiple harmful metabolic pathways, activate signaling molecules related to complications, and accelerate the occurrence of complications. When excessive fatty acids accumulate in the pancreas, it can impair beta-cell function and even induce apoptosis. When they deposit in peripheral target organs outside the pancreas, it can lead to secondary insulin resistance. High blood glucose not only mediates cell damage but also affects the synthesis and breakdown of fats, forming a vicious cycle with lipid metabolism and insulin resistance. Under normal circumstances, the brain, visceral organs, pancreas, and other insulin-target organs maintain glucose homeostasis by regulating the delicate balance between immune response and metabolism. In cases of disruption in lipid metabolism, macrophages in adipose tissue secrete inflammatory factors. This not only creates an inflammatory microenvironment within the pancreas, damaging the function of beta cells but also impairs the function and metabolic dynamics of various metabolic cells. It triggers a systemic chronic inflammatory response, thereby exacerbating the progression of T2DM and the occurrence of complications [49, 50]. In addition, the pro-inflammatory factors secreted by adipose tissue not only directly damage the vascular endothelium but also cause metabolic disorders, resulting in dysfunction of the vascular endothelium and promoting the formation of atherosclerosis [23]. Therefore, metabolic disorders of glucose and lipids can cause tissue-specific changes, leading to the occurrence and development of diabetes and its complications.

Of note, only a few studies have investigated the relative contributions of glucolipid metabolism markers to different complications and pancreatic function. The application of the TyG index, MHR, and TG/HDL in cluster analysis for newly diagnosed diabetes is also limited in relevant research. We found that 2hCP, FCP, FINS, FPG, TyG, HbA_1_C, and TG were correlated with HOMA2-β, while FINS, FPG, FCP, HbA1C and HDL were correlated with HOMA2-IR. Regarding the contributions of glucolipid metabolism markers to complications, FINS, TyG, 2hINS, and FCP showed differential superiority over other indicators. Cluster classification has promoted the progress of precision medicine, but clustering methods are often complex, requiring tedious calculation formulas and clustering software to categorize patients. This process needs further improvement to better guide clinical practice. Therefore, a classification pattern centered around pancreatic function may be more beneficial for the advancement of clinical work. Our study not only clarified the characteristics of cluster classification and the relative contributions of glycemic and lipid metabolism markers but also established a foundation for further improvements in classification methods.

## Study strengths and limitations

Our advantage lies in incorporating novel indicators that reflect pancreatic function and inflammatory factors and exploring the contributions of glucolipid metabolism markers to pancreatic function and complications. However, this is a single-center retrospective clinical study, future prospective studies and larger multicenter clinical studies should be conducted to clarify its causal relationship. In addition, due to limitations in conditions, indicators such as GADA, waist circumference, and visceral fat area were missing. Future studies should include the aforementioned indicators to further enhance the research.

## Conclusion

Insulin resistance was closely associated with inflammation and lipid metabolism disorders. 2hCP, FCP, FINS, FPG, TyG, HbA_1_C, HDL, and TG were correlated with the function of insulin. FINS, TyG, 2hINS, and FCP were associated with ASCVD, NAFLD, and CKD in newly diagnosed T2DM patients.

## Data Availability

The data analyzed during the current study is available from the corresponding author upon reasonable request.

## Abbreviations

SAID: Severe autoimmune diabetes
SIDD: Severe insulin-deficient diabetes
SIRD: Severe insulin-resistant diabetes
MOD: Mild obesity-related diabetes
TC: Total cholesterol
TG: Triglycerides
HDL: High-density lipoprotein
TyG: Triglyceride glucose index
FPG: Fasting plasma glucose
FINS: Fasting insulin
FCP: Fasting C-peptide
2hPG: 2-Hour postprandial plasma glucose
2hINS: 2-Hour postprandial insulin
2hCP: 2-Hour postprandial C-peptide
MHR: Monocyte/high-density lipoprotein ratio
NAFLD: Nonalcoholic fatty liver disease
ASCVD: Atherosclerotic cardiovascular disease
CKD: Chronic kidney disease
HOMA2-β: Homeostasis model assessment-2 beta-cell function
HOMA2-IR: Homeostasis model assessment-2 insulin resistance
